# Research Progress of Interstitial Lung Disease Based on Single‐Cell Sequencing Technology

**DOI:** 10.1155/carj/8863968

**Published:** 2026-02-02

**Authors:** Shujing Li, Tao Zhou, Jiying Zhang, Li Zhang, Qing Li, Bin Li, Jine Dai, Rui Yu, Yi Li, Shaoying Li

**Affiliations:** ^1^ 920th Hospital of Joint Logistics Support Force, PLA, Kunming, Yunnan, China; ^2^ Kunming Medical University, Kunming, Yunnan, China, kmmc.cn

**Keywords:** cell heterogeneity, idiopathic pulmonary fibrosis, interstitial lung disease, single-cell sequencing technology

## Abstract

Interstitial lung disease (ILD) refers to a collection of respiratory conditions characterized by inflammation of the lung parenchyma, alveolar irritation, and fibrosis of the interstitial tissue. Traditional research methods are often unable to completely reveal the complex mechanism of ILD occurrence and development. However, advancements in single‐cell sequencing technology in recent years have opened up a novel avenue for investigating ILD. This review summarizes recent single‐cell‐sequencing advances in the major interstitial lung diseases—idiopathic pulmonary fibrosis (IPF), pneumoconiosis, and connective tissue disease–associated ILD (CTD‐ILD)—and outlines future research priorities.

## 1. Introduction

**FIGURE 1:**
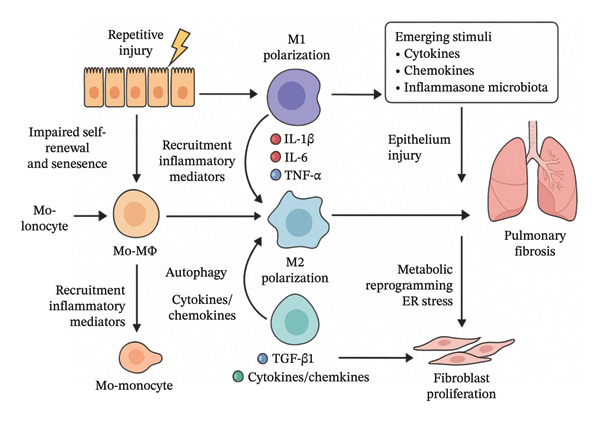
The interaction network of key pulmonary cells during the progression of pulmonary fibrosis (modified and drawn according to References [3–23]).

Interstitial lung disease (ILD) is characterized by interstitial inflammation, fibrosis, and destruction of alveolar structures, encompassing conditions such as idiopathic pulmonary fibrosis (IPF), pneumoconiosis, and connective tissue disease–associated ILD (CTD‐ILD) [1]. Given that the adult human body comprises approximately 37 trillion cells [2], each with distinct morphology, behavior, and function, deviations from normal cellular function may underlie many diseases. Different types of cells play distinct roles in the process of pulmonary fibrosis. The interactions between cells and the main signal transduction pathways are shown in Figure 1. Additionally, gene functions and mutations contribute to complex interactions within the cellular environment [24]. Traditional research methods, however, often fail to elucidate the roles of specific cell types and subpopulation heterogeneity in the complex pathogenesis of ILD. Conventional bulk RNA‐seq generates an averaged transcriptomic profile from pooled cells, thereby obscuring cell‐to‐cell gene‐expression differences and masking rare populations, subtle transcriptional shifts, and temporal dynamics [25]. Although flow cytometry has long been used for cell analysis, it cannot detect small‐molecule metabolites and is restricted to predefined protein targets, and cannot track dynamic changes at single‐cell resolution [26, 27]. Single‐cell RNA sequencing (scRNA‐seq) technology overcomes these limitations by resolving transcriptomes at the individual‐cell level, enabling the identification of key genes that regulate cell differentiation or maintenance. High‐throughput single‐cell sequencing, therefore, provides new opportunities to clarify the pathogenesis of ILD and to advance prognostic evaluation and personalized treatment.

## 2. Effect of Lung Cell Heterogeneity on ILD

The lung is a complex organ composed of many different cell types. Cellular and molecular factors together constitute the immune regulatory network, which has a significant impact on lung tissue health. Studies have found that in the lung tissues of IPF patients, the number of fibrotic and inflammatory cells is significantly increased. The distribution of epithelial cells and immune cells also differs from that in healthy tissues. Mononuclear macrophage cell lines, granulocytes, lymphocytes, mast cells, and other cell types play important roles in the development of IPF [28]. Specific transcriptomic changes and signaling pathway activation in these cell subpopulations suggest a close link between chronic inflammatory responses, immune dysregulation, and fibrotic processes.

### 2.1. Alveolar Macrophages

Interstitial macrophages, TR‐AMs, and monocyte‐derived alveolar macrophages (Mo‐AMs) are the primary macrophage populations in the lung [3–7]. TR‐AMs serve as outposts for maintaining immune homeostasis, whereas the characteristics and functions of Mo‐AMs are predominantly shaped by the pulmonary microenvironment [8]. Collectively or individually, these macrophage subsets contribute to homeostasis maintenance, immune surveillance, phagocytosis, and inflammation resolution. They also interact with each other to preserve immune balance [9]. Research has demonstrated that TR‐AMs can self‐renew independently of monocyte proliferation under steady‐state conditions. However, TR‐AMs may be depleted following severe insults such as ionizing radiation or viral infection [8, 18, 19, 21]^,^. Depending on the extent of damage, AMs can be replenished either through the local proliferation of residual TR‐AMs [18] or by recruiting monocytes into the alveolar niche to differentiate into Mo‐AMs [19, 21, 22]. Macrophages of different origins can coexist within the lung. Newly recruited Mo‐AMs exhibit greater plasticity compared with TR‐AMs. The differentiation of Mo‐AMs is regulated by cytokines and metabolites in the local environment. These signals induce the expression of different transcription factors, thereby affecting the function of specific macrophages [10–13]. During the process of fibrosis, AMs become partially exhausted and are replaced by Mo‐AMs [21, 23], which can be selectively deleted to improve fibrosis [19]. These studies suggest that monocyte‐derived cells are more likely to differentiate into profibrotic phenotypes and promote fibrotic processes. However, the interaction between the fibrotic environment and Mo‐AMs differentiation remains unclear. Collectively, the evidence demonstrates that Mo‐AMs adopt a pronounced profibrotic phenotype during the evolution of pulmonary fibrosis. Importantly, this phenotypic plasticity is not an autonomous process but is critically instructed by stromal cells within the fibrotic niche. Fibroblasts—the principal effectors of extracellular‐matrix (ECM) deposition—exhibit spatiotemporal synchrony with the functional reprogramming of discrete macrophage subsets. Through paracrine release of key mediators such as TGF‐β, fibroblasts actively remodel the immune microenvironment; reciprocally, cytokines elaborated by immune cells potentiate fibroblast activation, thereby perpetuating a deleterious feed‐forward loop. Consequently, high‐resolution characterization of fibroblast heterogeneity at the inception phase of fibrosis represents a pivotal gateway to elucidating the profibrotic role of Mo‐AMs.

### 2.2. Epithelial Cell

Although stromal cells, epithelial cells, and immune cells are all involved in the development of pulmonary fibrosis, the first published study using scRNA‐seq to investigate the pathobiology of pulmonary fibrosis focused on epithelial cells [14]. For example, Strunz and colleagues performed scRNA‐seq on lung tissue from mice with bleomycin‐induced pulmonary fibrosis [15], identifying and validating a new progenitor cell population of Krt8+ transitional epithelial cells that significantly increase in number during the early stages of pulmonary fibrosis. Simultaneously, RNA velocity analysis revealed that the transcriptional states of airway stem cells, alveolar type II (AT2) cells, and club cells converge toward the Krt8+ state, ultimately differentiating into alveolar type I (AT1) epithelial cells. During this process, the TNF‐α/NF‐κB pathway, p53, and hypoxia predominantly drive differentiation over Sox4, Ctnnb1, and Wwtr1. Schiller et al. confirmed that epithelial cell plasticity can induce nonprogressive transdifferentiation. However, if the checkpoint signals of terminal differentiation are disrupted, these intermediate states may persist and potentially promote fibrosis. Collectively, recent work implicates epithelial plasticity—particularly the emergence of Krt8^+^ transitional cells—in initiating fibrosis. What remains unclear is how derailed terminal‐differentiation checkpoints precipitate pathological activation of interstitial effector cells. Fibroblasts are the principal drivers of matrix remodeling, and their phenotypic switching is tightly governed by epithelial–mesenchymal crosstalk: aberrantly differentiated epithelia secrete TGF‐β and related mediators that activate neighboring fibroblasts, while the resulting ECM deposition, in turn, perturbs epithelial homeostasis. Consequently, high‐resolution mapping of fibroblast heterogeneity, with emphasis on their earliest transcriptional and functional responses to epithelial injury, is now pivotal to dissecting the fibrotic cascade.

### 2.3. Fibroblast Cell

Fibroblasts are mesenchymal cells that contribute to tissue homeostasis and remodeling through proliferation, migration, transdifferentiation, and ECM production [16]. Mayr et al. utilized a combination of spatial transcriptomics sequencing (st‐seq), multiple immunostaining, longitudinal RNA sequencing (RNA‐seq), and genetic lineage tracking to identify transition‐state fibroblasts characterized by high SFRP1 expression. SFRP1‐expressing cells emerge early after injury in peribronchiolar, pleural, and alveolar regions, preceding myofibroblasts. SFRP1 regulates TGF‐β1‐induced fibroblast invasion and RhoA pathway activity. SFRP1 inhibits fibroblast invasion during fibrogenesis and may limit or reverse fibroblast formation [29]. In summary, epithelial injury initiates a tricellular cascade: injured epithelium recruits monocytes and instructs their differentiation into profibrotic macrophages; these macrophages secrete factors that activate fibroblasts; activated fibroblasts, in turn, modulate macrophage behavior and epithelial repair. Understanding these sequential and reciprocal interactions provides a unified framework for targeting fibrosis at multiple cellular checkpoints.

## 3. Application of sc‐RNA Seq in ILD Research

### 3.1. Exploring the Pathological Mechanism of ILD

ScRNA‐seq enables the monitoring of transcriptomic changes in individual cells throughout the progression of ILD, thereby facilitating the identification of key pathogenic mechanisms and signaling pathways. For instance, a specific subtype of macrophages or epithelial cells that exhibit abnormal gene expression is associated with epithelial–mesenchymal transition, fibrosis, and immune‐mediated inflammation, all of which contribute to lesion development. These findings can aid in our understanding of the pathogenesis of ILD at the cellular and molecular levels and in the search for effective therapeutic targets.

#### 3.1.1. IPF

IPF is a disease that ultimately leads to pulmonary fibrosis due to diffuse alveolar inflammation and an abnormal alveolar structure. In clinical practice, it accounts for 20% of all ILD cases. The reported prevalence of IPF varies, ranging from 0.7% (0.1 million people) in Taiwan to 63.0% (0.1 million people) in the United States [30]. The reported incidence rate ranges from 0.6 to 17.4 per 100,000 person–years [31]. It has gradually been discovered that its pathogenesis has shifted from being fibroblast‐driven to being epithelial cell‐driven [32]. Adams et al. studied 312,928 cells from the lungs of patients with IPF, smokers and nonsmokers (as controls), and patients with chronic obstructive pulmonary disease (COPD). They identified 18 immune cell subpopulations, including profibrotic macrophage subpopulations. In addition, the study also found that there are some abnormal basal‐like cells in the edge area of myofibroblast foci. These cells simultaneously express basal epithelial, mesenchymal, aging, and developmental marker genes. There is also heterotopic proliferation of endothelial cell subpopulations. Furthermore, there is a partial overlap between myofibroblasts and invasive fibroblasts in the control group and in patients with COPD [33]. Researchers have also found that in the early stages of IPF development, epithelial cells exhibit the upregulation of cell proliferation‐related genes and signs of epithelial–mesenchymal transition [34, 35]. Habermann et al. performed scRNA‐seq on single‐cell suspensions from 10 nonfibrotic lungs and 20 fibrotic lungs, identifying a total of 31 different cell subpopulations. Among them, epithelial cell differentiation showed significant differences. Specifically, there were the highly concentrated KRT5/KRT17 pathological subtype and ECM‐producing epithelial subtype in fibrotic lung tissue. These were previously unrecognized epithelial cell phenotypes. In addition, multiple fibroblast subtypes promoted ECM accumulation in a spatially discrete manner [36]. In summary, these research findings provide us with a new perspective for a deeper understanding of the pathological mechanisms of pulmonary fibrosis and COPD.

In summary, the traditional paradigm posited that IPF is primarily inflammatory; however, the consistent failure of immunosuppressive regimens argues against inflammation as the principal driver. Current consensus instead positions repeated alveolar epithelial cell injury as the initiating event, with cellular senescence, endoplasmic‐reticulum stress, and mitochondrial dysfunction collectively precipitating maladaptive repair and progressive fibrosis [37]. Activated fibroblasts subsequently elaborate ECM proteins in response to AEC‐derived cues, yet their precise ontogeny and mechanistic contribution remain contentious. Recent scRNA‐seq analyses have identified a pathogenic basal‐like epithelial population (KRT5^+^/KRT8^+^) [36] in IPF lungs that appears to orchestrate fibrosis directly. Thus, while epithelial–fibroblast crosstalk is evident, epithelial cells emerge as the principal instigators, with fibroblasts acting as effectors rather than drivers.

#### 3.1.2. Pneumoconiosis

Pneumoconiosis is a disease characterized by diffuse fibrosis of lung tissue, primarily caused by the long‐term inhalation of dust. From 1990 to 2017, the number of pneumoconiosis cases in both males and females increased by 81.1%. The incidence rate rose with age, and there were more male patients than female patients [38].

Peng et al. conducted scRNA‐seq on BALF samples from miners with silicosis and their colleagues without the disease, revealing that impaired interferon‐γ (IFN‐γ) signaling in bone marrow cells is closely linked to the development of silicosis [39]. In vitro experiments confirmed that IFN‐γ‐induced spacious phagosome formation in macrophages inhibits silica‐induced lysosomal membrane damage by reducing the ratio of silica/phagosome area to volume, thereby alleviating lung injury. Their other study revealed that RAB20 deficiency in monocytes/macrophages is closely related to the development of silicosis. In a murine model of silicosis, RAB20 knockout enhanced IL‐1β release and NLRP3 inflammasome activation, significantly aggravating silica‐induced interstitial fibrosis and respiratory dysfunction [40]. In an in vitro model, RAB20‐knockout macrophages enhanced the release of IL‐1β and NLRP3 inflammasome activation induced by crystalline silica. This was partly due to an elevated ratio of crystalline silica/phagosome area to volume, leading to lysosomal membrane damage. These findings provide novel molecular insights into the lysosomal damage and rupture caused by macrophage phagocytosis of silica crystals, suggesting that targeting lysosomal rupture and cell damage may delay the progression of pneumoconiosis.

Yang et al. discovered that a glycoprotein called glycoprotein nonmetastatic melanoma protein B (GPNMB) on AT2 epithelial cells may induce epithelial‐to‐mesenchymal transition by accelerating cell proliferation and migration, as well as increasing mesenchymal markers, ultimately leading to sustained lung damage [41]. Additionally, GPNMB is released in the form of extracellular vesicles to participate in the epithelial–mesenchymal transition of distant epithelial cells, thereby accelerating the development of silicosis. Meanwhile, abnormal changes in the composition of the extracellular matrix and collagen structure can promote vesicle adhesion, further accelerating the development of fibrosis. Moreover, streptomycin may achieve the goal of inhibiting fibrosis by inhibiting the extracellular N‐glycosylation of GPNMB‐expressing cells.

Joshi et al. used scRNA‐seq and spatial transcriptomics techniques to study pulmonary fibrosis tissues in mice and humans. They found a type of macrophage located near fibroblasts, expressing colony‐stimulating factor (M‐CSF) that can maintain their differentiation state. Simultaneously, the expressed platelet‐derived growth factor subunit A (PDGF‐A) can promote fibroblast proliferation. Blocking M‐CSF may inhibit the differentiation of profibrotic macrophages and thus suppress fibrosis [31]. This study suggests that the M‐CSF signaling pathway may be a potential target for controlling the differentiation and maintenance of profibrotic macrophages.

#### 3.1.3. Connective Tissue Disease–Related ILD

Valenzi et al. also performed scRNA‐seq on 13 explanted lung tissue samples from four healthy controls and four patients with systemic sclerosis–associated interstitial lung disease (SSc‐ILD). They found that the phenotype of myofibroblasts was significantly altered compared with that of healthy individuals, with a significant increase in collagen production and other profibrotic genes. These findings suggest that myofibroblast differentiation and proliferation may represent a key cell population driving fibrosis in SSc‐ILD [42]. Therefore, scRNA‐seq can identify cell subpopulations with high expression levels of profibrotic genes and accurately reveal differences in fibrotic gene expression among cells from different patients, which can guide clinical diagnosis and treatment.

### 3.2. The Regulatory Mechanism of Lung Microenvironment

The application of scRNA‐seq technology has enabled us to conduct in‐depth research on the cell populations that affect the lung microenvironment in ILD, including the regulation of interactions and signaling pathways between immune cells in the lungs. Mu et al. found that the alveolar structure and immune microenvironment of mice exposed to coal dust were disrupted. The pathways of inflammation and cell death (apoptosis, autophagy, and necrosis) were activated, thereby mediating the occurrence of pneumoconiosis [43]. During the development process, AT2 cells exhibit abnormal proliferation and high activity. The expression of LYZ and Chi3l1 antibacterial proteins decreases, leading to a lack of antibacterial function. A new subset of macrophages with M2‐polarized double expression of MLPH+/CD206+ was identified in mice with pneumoconiosis. However, this cell subset was significantly reduced after vitamin D treatment. It was also found that the activation of the hypoxia/Notch signaling pathway in epithelial cells interacts with genes controlling movement and invasion in macrophages. In summary, these findings provide potential targets for the treatment of pneumoconiosis.

## 4. Single Cell Sequencing Provides New Ideas for ILD Treatment

### 4.1. Therapeutic Strategies Targeting Subpopulations of Cells

The advancement of single‐cell sequencing technology has enabled researchers to gain a better understanding of the molecular characteristics and functions of different cell subpopulations in ILD. This, in turn, provides new ideas for developing therapeutic strategies that target specific cell types or subpopulations. Xu et al. found that cells in IPF often coexpress AT1, AT2, and airway‐selective markers. These cells exhibit an “uncertain” differentiation state that is not observed during normal lung development. They identified three distinct subsets of epithelial cells with airway basal and goblet cell features, as well as an atypical transitional cell population. This population promotes the pathological process of fibrosis and suggests potential interactions between various clusters of lung epithelial cells [44]. These findings indicate that strategies for treating IPF may need to target specific drug‐sensitive cell subpopulations or interaction pathways.

### 4.2. Identification of Potential Drug Targets Using Single‐Cell Transcriptome Analysis

Single‐cell sequencing technology can also be used to identify key transcription factors and signaling pathways involved in the development of ILD, thereby helping to discover potential drug targets. Aran et al. identified a transitional fibrotic macrophage subpopulation (CX3CR1+ SiglecF+), which is located at the site of accumulation of PDGFRa+ and PDGFRb+ fibroblasts. This subpopulation may induce pulmonary fibrosis by promoting fibroblast migration and proliferation and may serve as a new target for pulmonary fibrosis treatment [45]. Additionally, one of the FDA‐approved therapies for pulmonary fibrosis is the small molecule receptor tyrosine kinase inhibitor nintedanib, which blocks PDGF receptor signaling and delays fibrosis progression [46].

### 4.3. The Prospect of Individualized Treatment

By combining single‐cell data with clinical data, we can more accurately predict the occurrence and development of diseases. This allows for earlier intervention and treatment in patients and guides the implementation of personalized diagnosis and treatment. Tang et al. found that human umbilical cord mesenchymal stem cells (hucMSCs) can improve pulmonary fibrosis in mice. Through scRNA‐seq, a subpopulation of macrophages with interferon‐specific sensitivity was further identified. It was further confirmed that hucMSCs may improve pulmonary fibrosis by inducing macrophage expression of CXCL10, recruiting regulatory T cells to suppress inflammation, and thus achieving the goal of improving pulmonary fibrosis [47]. This discovery provides guidance for the clinical application of stem cell therapy for IPF and offers strong theoretical support for future applications.

## 5. The Limitations of scRNA‐seq

ScRNA‐seq has markedly advanced our appreciation of cellular heterogeneity, yet its intrinsic limitations remain pertinent. Batch effects frequently drive sample clustering by technical provenance rather than biological identity, obscuring genuine intercell variation. Dropout events introduce an excess of nonbiological zeros, yielding bimodal or skewed expression profiles that confound downstream clustering and differential analysis. Additionally, amplification bias disproportionately enriches select transcripts, embedding systematic errors that are difficult to rectify [48]. As public datasets expand exponentially, the absence of a unified, interoperable framework for cell‐type annotation and nomenclature continues to impede the direct translation of single‐cell atlases into clinically relevant insights. Moreover, scRNA‐seq delivers only a static, “snapshot” transcriptome and, therefore, cannot capture the immune system’s dynamic cascade from genome to phenome via transcript and protein layers [49]. Consequently, future studies must integrate complementary multiomic and spatiotemporal approaches to fully elucidate the pathobiology of pulmonary fibrosis.

## 6. Conclusion

ScRNA‐seq technology has become an important tool for revealing the cellular composition and transcriptome expression profiles at ILD lesion sites. In the future, with the continuous development of technology, it will continue to play an important role in ILD pathogenesis research, prognosis evaluation, and personalized treatment. Among these, the following directions are particularly noteworthy: (1) further optimizing single‐cell separation and sequencing technology to improve detection sensitivity and accuracy; (2) integrating multiple omics data and combining clinical information to explore more refined pathological subtyping and personalized treatment strategies; and (3) future research needs to further investigate the infiltration mechanisms and functions of immune cells in lung tissue, reveal their specific roles in the occurrence and development of lung diseases, and provide a theoretical basis for the diagnosis, treatment, and intervention of lung tissue diseases.

**Nomenclature**
CTD‐ILD: Connective tissue disease–associated interstitial lung disease
ILD: Interstitial lung disease
IPF: Idiopathic pulmonary fibrosis
scRNA‐seq: Single‐cell RNA sequencing

## Author Contributions

Concept or design: Shujing Li and Shaoying Li.

Literature integration analysis: all authors.

Drafting of the manuscript: Shujing Li and Shaoying Li.

Critical revision of the manuscript for important intellectual content: all authors.

## Funding

This review was supported by the Yunnan Province Young and Middle Aged Academic and Technical Reserve Talent Project Fund (no. 202005AC160054).

## Disclosure

All authors had full access to the data, contributed to the study, approved the final version for publication, and take responsibility for its accuracy and integrity.

## Ethics Statement

This article does not involve ethical approval or other issues.

## Conflicts of Interest

The authors declare no conflicts of interest.
